# Effects of Laser Spot Size on the Mechanical Properties of AISI 420 Stainless Steel Fabricated by Selective Laser Melting

**DOI:** 10.3390/ma14164593

**Published:** 2021-08-16

**Authors:** Xi-Huai Yang, Chong-Ming Jiang, Jeng-Rong Ho, Pi-Cheng Tung, Chih-Kuang Lin

**Affiliations:** Department of Mechanical Engineering, National Central University, Jhong-Li District, Tao-Yuan City 32001, Taiwan; gjacky7205@gmail.com (X.-H.Y.); bcc750627@gmail.com (C.-M.J.); jrho@ncu.edu.tw (J.-R.H.); t331166@ncu.edu.tw (P.-C.T.)

**Keywords:** selective laser melting, AISI 420 stainless steel, laser spot size, mechanical properties

## Abstract

The purpose of this study is to investigate the effects of laser spot size on the mechanical properties of AISI 420 stainless steel, fabricated by selective laser melting (SLM), process. Tensile specimens were built directly via the SLM process, using various laser spot diameters, namely 0.1, 0.2, 0.3, and 0.4 mm. The corresponding volumetric energy density (*E_V_*) is 80, 40, 26.7, and 20 J/mm^3^, respectively. Experimental results indicate that laser spot size is an important process parameter and has significant effects on the surface roughness, hardness, density, tensile strength, and microstructure of the SLM AISI 420 builds. A large laser spot with low volumetric energy density results in balling, un-overlapped defects, a large re-heated zone, and a large sub-grain size. As a result, SLM specimens fabricated by the largest laser spot diameter of 0.4 mm exhibit the roughest surface, lowest densification, and lowest ultimate tensile strength. To ensure complete melting of the powder and melt pool stability, *E_V_* of 80 J/mm^3^ proves to be a suitable laser energy density value for the given SLM processing and material system.

## 1. Introduction

Selective laser melting (SLM) is one of the popular additive manufacturing (AM) techniques in fabricating metallic components [1,2]. SLM is a powder-bed fusion process, in which a laser beam is employed to locally melt the feedstock of metallic powder and join materials in a layer-by-layer manner [3]. The SLM process produces complete melting and forming on the powder bed, so a high density (close to 100%) can be achieved with an optimized combination of the process parameters. The final quality of the SLM build is significantly dependent on the heat input, which is controlled by the laser process parameters. There are numerous studies investigating the correlation between process parameters (e.g., laser heat input, scanning strategy, and build direction) and the SLM build qualities (such as density, surface roughness, and mechanical properties) [3,4,5,6,7,8]. The amount of energy per unit volume, which is called energy density, is associated with the SLM process parameters, such as laser power (*P*), laser scanning speed (*v*), layer thickness (*t*), laser spot diameter (*d*), hatch distance (*h*), stripe width (*w*), and stripe overlap (*r*) [4]. A parameter combination, namely volumetric energy density (*E_V_*), is a common metric for SLM processes [9,10]. *E_V_* is a combination of laser power, laser scanning speed, layer thickness, and laser spot diameter and is defined as *E_V_* = *P*/(*v·d·t*) [9,10].

Given various *E_V_* values, there exists a critical value (*E_C_*) of SLM process for each material [5]. In an SLM process with constant *E_C_*, the scanning path is continuous, with a constant width of stable melt pool, such that the as-deposited path can be well-bonded to the baseplate or the previous layer [4]. When the laser energy density is lower than *E_C_* with insufficient heat input, the incompletely melted powder will generate voids inside the SLM builds [4]. In addition, the melt pool instabilities result in a “balling” phenomenon on the laser scanning track, which increases surface roughness [4,11]. On the other hand, when the laser energy density is higher than *E_C_*, a “keyhole” melting mode of a very deep melt pool is generated [4,12,13]. The overheated, high-temperature condition also causes some over-melted powder to splash out, forming several attachments around the laser scanning track [4,12,13]. The place where splashing attachments occur is random and it is different from the balling on the laser scanning track. Therefore, for certain applications, the SLM process parameters can be optimized by adjusting the laser energy density (*E_V_*) to achieve high build rate, low porosity, high geometrical precision, and low surface roughness.

One way to improve the build rate of the SLM process is increasing the laser power, which allows for higher scanning speed, larger laser spot size and hatch spacing, and greater layer thickness [14,15,16]. Among the SLM process parameters, the spot size of the laser is an effective variable in controlling the laser energy density, so as to adequately melt the powder and build parts of acceptable quality [17]. The appropriate laser spot size should provide sufficient energy density to melt the current powder layer and fuse to the previously melted one, but also to be small enough to meet requirements of the dimensional precision and surface roughness [17]. The effects of laser spot size, on various characteristics of SLM builds, have been investigated by a limited number of studies [17,18,19,20,21,22]. Change of laser spot size, through focal shift, would affect the laser energy density, porosity, and microstructure of the Inconel 718 alloy; a finer microstructure is found for a smaller laser spot [17,18]. In the study of Shi et al. [19], it is concluded that a larger laser beam diameter is more suitable for high-power SLM in fabricating Ti-6Al-4V parts with thick layers. By changing the laser spot size via varying defocus distance, a significant influence on the size and morphology of the melt pool is found in producing 316L stainless steel by SLM [20]. It is found that a good surface finish in SLM builds of AlSi12 alloy can be obtained with an optimal combination of defocus distance and laser power [21]. In the study of SLM builds of IN 625 alloy [22], the surface roughness is increased, with increasing laser spot diameter, but the tensile strength is barely affected by the laser spot size.

AISI 420 stainless steel is one of the martensitic steels. It has an excellent combination of mechanical properties and corrosion resistance, so it is wildly used in various industries [23]. In recent years, using AM techniques to fabricate molds with complex conformal cooling channel becomes more and more popular. SLM is a favorite process in fabricating a mold with conformal cooling channel, and AISI 420 is suitable for such application, due to its low cost and high absorption of laser radiation [8]. However, only a few studies have been focused on the characteristics of AISI 420 fabricated by SLM [8,24,25,26]. In order to be applicable to the plastic injection mold, a SLM process window of laser power and scanning speed has been obtained for AISI 420, with a high density and hardness [8]. Effects of build direction on the mechanical properties of AISI 420, fabricated by SLM, have been studied by Shen et al. [26]. Saeidi et al. [24] and Krakhmalev et al. [25] have investigated the effects of heat treatment on the microstructure and mechanical properties of SLM builds of AISI 420. As described above, laser spot size plays in important role in determining the density, surface, microstructure, and performance of metallic SLM builds [17,18,19,20,21,22]. However, there is lack of study on the effects of laser spot size on the microstructure, surface finish, and mechanical properties of AISI 420 fabricated via SLM. As an extension of our earlier work [26], the aim of this study is to investigate the relationship of the spot size with the surface finish and mechanical properties of SLM built AISI 420 parts. The results of this study, and our previous study [26], can hopefully contribute to improving the quality of the SLM builds of AISI 420, for applications in the plastic injection mold and other industries.

## 2. Experimental Procedures

### 2.1. Specimen Fabrication

For tensile test purposes, plate-type tensile specimens of dog-bone shape were directly built by SLM process to the final dimensions. Dimensions of gauge section, in the tensile specimen, were of 32 mm (length) × 6 mm (width) × 3 mm (thickness). Detailed geometry and dimensions of the tensile specimen were given in our earlier study [26]. The AISI 420 stainless steel powder (Sanyo Special Steel Corporation, Ltd., Hyōgo-ken, Japan) is supplied with a particle size, ranging from 10 μm to 88 μm. The major elements in chemical composition include 13.04 wt% Cr, 0.355 wt% C, 0.59 wt% Si, 0.36 wt% Mn, 0.26 wt% Ni, and balance of Fe, as per vendor’s data sheet. A commercial laser powder-bed SLM machine (LPM325, Sodick Corporation, Ltd., Kanagawa, Japan), equipped with an ytterbium-doped fiber laser, is employed to fabricate the tensile specimens.

**Figure 1:**
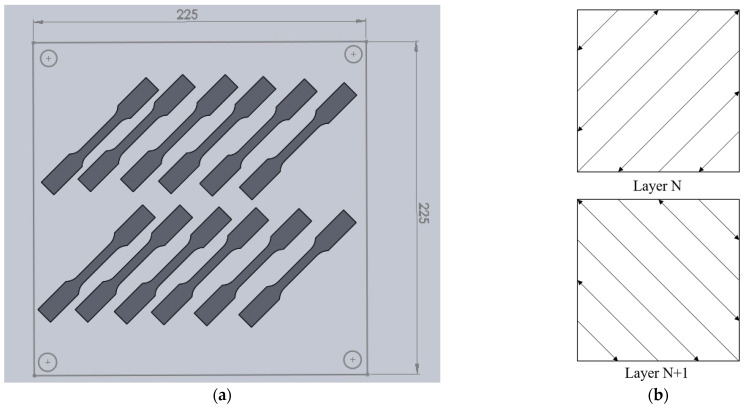
Schematic of (**a**) specimen placement (dimensions in mm) and (**b**) layer-related scanning strategy.

The SLM process parameters and scanning strategy are shown in Figure 1 and Table 1. The layer-related scanning strategy follows the default setting of 45° and 135° rotation scanning in the SLM machine, as shown in Figure 1. In order to make the scanning path parallel or perpendicular to the longitudinal axis of the tensile specimen in alternate layers, the longitudinal axis of the tensile specimen is rotated 45° from the horizontal orientation of the baseplate, as shown in Figure 1. For the pattern-related scanning strategy on a given layer, an island pattern with alternating path is used. Twelve tensile specimens were fabricated, using various laser spot sizes, in a single batch, by the given SLM machine, as shown in Figure 1. The SLM builds were cut off from the baseplate by wire electrical discharge machining after laser process. The given SLM process parameters are listed in Table 1. The 12 tensile specimens were divided into four groups, namely S1, S2, S3, and S4, with various laser spot diameters. Each group has 3 specimens of the same process parameters and scanning strategy. In other words, three tensile tests were repeatedly conducted for each given laser processing condition. The laser spot diameters were 0.1 mm, 0.2 mm, 0.3 mm, and 0.4 mm for S1, S2, S3, and S4, respectively. To investigate the inherent effects of laser spot size on the surface roughness, microstructure, and mechanical properties, the SLM specimens were kept in the as-built condition. Therefore, no post-process treatments, such as machining, polishing, or heat treatment, were applied to the specimens.

### 2.2. Measurement of Surface Roughness, Density, and Hardness

Surface morphology was observed and measured by a 3D laser scanning confocal microscope (VK-9710, Keyence Corporation, Osaka, Japan), which is a non-contact technique to determine the surface roughness. Its image resolution is 0.001 μm. Two types of surface roughness were measured, namely average surface roughness of the profile (*R_a_*) and maximum surface roughness (*R_max_*). Five regions in the gauge section of each specimen were selected for the surface roughness measurement, because it was expected that fracture will occur at the gauge section of specimen. The 3D surface morphology was also used to determine the line surface roughness in the length*-*and width*-*directions. Density of the SLM builds was measured by Archimedes’ principle. The relative density of the SLM specimen is defined as *ρ*_r_ = *ρ*_sample_/*ρ*_reference_. *ρ*_sample_ represents the density of the specimen, and the reference density of AISI 420 steel was given as *ρ*_reference_ = 7.80 g/cm^3^ [27]. Hardness of the SLM builds, in Rockwell C scale, was measured using a hardness tester (AR-10, Akashi Corporation, Osaka, Japan). Ten different places in the grip section were selected for hardness measurement for each tensile specimen. 

### 2.3. Tensile Test

A commercial servo-hydraulic material test machine (MTS 810, MTS System Corporation, Eden Prairie, MN, USA) was employed to conduct the tensile test, for determining the tensile properties, including elastic modulus, yield stress, ultimate tensile stress, and elongation. The tensile test was performed under displacement control, with a stroke rate of 0.5 mm/min. The strain during testing was measured using a uniaxial extensometer (MTS 634.12F-24, MTS System Corporation, Eden Prairie, MN, USA). 

### 2.4. Fractography and Microstructural Analysis

Metallographic and fractographic analyses were applied for investigating microstructure and fracture pattern of the SLM built specimens. Fracture surfaces were observed using scanning electron microscopy (SEM, Hitachi S-800, Hitachi, Ltd., Tokyo, Japan) to characterize the fracture pattern and find the fracture origin. In addition to SEM, optical microscopy (OM, BX51M, Olympus Corporation, Tokyo, Japan) was also employed to analyze the microstructure. For microstructural analysis, the metallographic samples were ground with sandpapers and polished using 0.3-μm and 1-μm Al_2_O_3_ paste. After that, the metallographic samples were chemically etched in an acidic solution of 2% HF and 8% HNO_3_. Energy dispersive spectrometer (EDS) was also utilized to identify the chemical composition at specific positions. X-ray diffraction (XRD) analysis was applied to determine the crystalline phases in the given SLM specimens. Details of the XRD analysis technique were given elsewhere [26].

## 3. Results and Discussion

### 3.1. Surface Roughness

**Figure 2:**
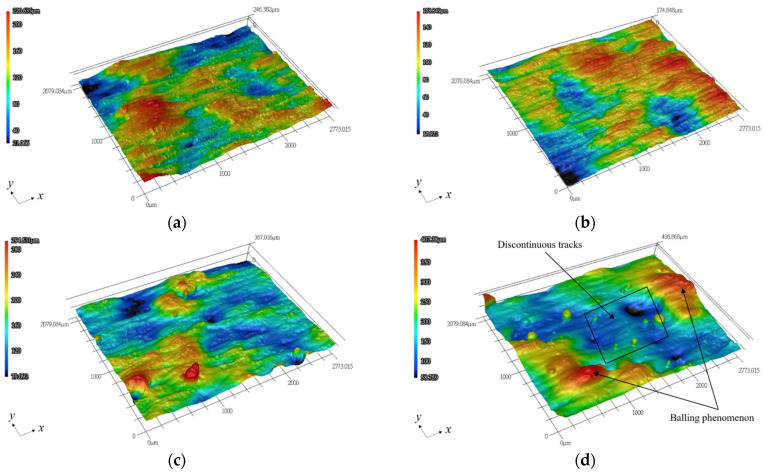
Representative 3D profiles of the top surface, measured by a laser scanning confocal microscope: (**a**) S1; (**b**) S2; (**c**) S3; (**d**) S4.

Figure 2 shows the typical morphology of the top surface of final layer in each group, observed by the laser scanning confocal microscope. Measurements of surface roughness, in a line along the *x* (length) and *y* (width) directions, respectively, denoted by *R_x_* and *R_y_*, are shown in Table 2. Note that *R_x_* was measured along the laser scanning direction, while *R_y_* was measured in the direction perpendicular to the laser scanning path. As shown in Table 2, the average values (*R_a_*) of *R_x_* in S1, S2, and S3 are comparable, and their standard deviation is very small. However, the *R_a_* of *R_x_* in S4 is significantly greater than that of the other three groups. On the other hand, the difference in *R_a_* of *R_y_* for the given four groups is insignificant, but the standard deviation is significantly larger than that of *R_x_*. The surface roughness along the scanning direction is apparently smoother than that in the transverse direction, in particular for S1, S2, and S3. The main reason is that the *R_y_* was measured in the direction across multiple scanning tracks. However, this is not the case for S4, as its average value of *R_x_* is even larger than that of *R_y_*. This is attributed to the occurrence of severe balling in S4, as shown in Figure 2d. In particular, the maximum roughness value (*R_max_*), which may be regarded as an index of the ball size, generally increases with increasing laser spot size. A noticeable difference in the *R_max_* values of both *R_x_* and *R_y_* was found among the given laser spot sizes. This is evidenced by the 3D surface profiles, shown in Figure 2. As shown in Figure 2c,d, balling occurs in both S3 and S4 specimens, particularly, to a greater extent in the latter. As shown in Figure 2d, balling occurred when the scanning path tended to split into several spherical balls and turned into discontinuous tracks. Therefore, the rougher surface, fabricated by a larger laser spot size, is attributed to the balling effect, caused by a lower volumetric energy density. When the laser energy density was too low to fully melt the powder, the wetting effect deteriorated, and the balling effect occurred by adhering large balls to the track. As listed in Table 1, *E_V_* is 80, 40, 26.7, and 20 J/mm^3^ for S1, S2, S3, and S4, respectively. As serious balling is barely seen in S1 and S2, the critical volumetric energy density for the given SLM system is likely within the range of 26.7–40 J/mm^3^.

### 3.2. Density and Hardness

Table 3 shows the density measurements for the given samples, with a very good repeatability of density data in each group. The average relative density (*ρ_r_*) ranges from 91% to 97% for the SLM samples. The *ρ_r_* generally decreases with an increase in laser spot size, revealing that Group S4 has the largest porosity ratio. The *ρ_r_* in S1, S2, and S3 is greater than 95%, while it is only 91% in S4. It is expected that a larger number of pores formed, due to an improper joining between two layers in a lower laser energy density. Therefore, more pores were generated at the edge of the melt pools in Group S4 and reduced the density, as compared to the other groups. Variations of the relative density and porosity ratio, with respect to laser spot size, can be related to the volumetric energy density. Comparison of Groups S1 to S4 indicates the relative density increases from 91% to 97%, in line with a lower fraction of porosity generated by a smaller laser spot size with higher volumetric energy density. Note that the *E_V_* applied for fabricating S4 specimens is 20 J/mm^3^, which is only a quarter of 80 J/mm^3^ for Group S1. Details of microstructural observation of pores in the given SLM builds are given in Section 3.5.

Measurements of hardness are also shown in Table 3. The hardness of each specimen (e.g., S1-1) is represented by the average and standard deviations of 10 measurements, and the average of all the measurements in the same group (e.g., S1-1 to S1-3) is taken to represent the hardness of each group (e.g., Group S1). The average HRC of S1 is the highest, followed by S2, S3, and S4. The AISI 420 stainless steel, fabricated by conventional processes, generally has a hardness around 50 HRC [28]. The given as-built SLM AISI 420 parts exhibits a higher hardness (56–62 HRC), compared to that fabricated by conventional processes. In comparison of Groups S1 to S4, it is found that the specimens built by a smaller laser spot, with a higher energy density, have a higher density, leading to a higher hardness. Therefore, there is a good correlation between the density and hardness measurements. This is also consistent with the trend that S1 specimens with the lowest porosity ratio also possesses the highest tensile strength among the given laser spot sizes, as described below.

### 3.3. Tensile Properties

**Figure 3:**
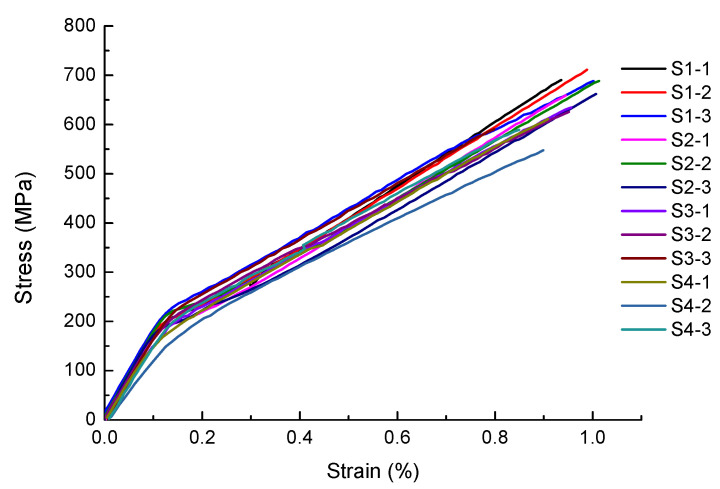
Stress-strain curves of SLM AISI 420 built with various laser spot sizes.

Figure 3 shows the stress-strain curves of Groups S1 to S4. As shown in Figure 3, all the elongation is around 1%, indicating the as-fabricated SLM builds of AISI 420 stainless steel are brittle ones. It seems that each stress-strain curve behaves in a bi-linear manner, as shown in Figure 3. The initial linear elastic region of each stress-strain curve was used to calculate the Young’s modulus. After yielding, the stress-strain curves still show a linear relationship. The bi-linear stress-strain curve may be attributed to phase transformation of unstable retained austenite to martensite during tensile testing. Due to the thermodynamic instability in the SLM process, a certain amount of austenite was retained in the microstructure of as-built AISI 420 stainless steel [26]. During loading or deformation, unstable retained austenite may transform to martensite [29]. Therefore, the transformation-induced plastic strain was generated during the tensile testing [29]. The turning point at each bi-linear stress-strain curve is defined as the yield stress. Table 4 lists the tensile properties of each as-built specimen. The average Young’s modulus of S1 to S4 is 176.2, 178.3, 174.9, and 155.4 GPa, respectively. For Group S4, the lower Young’s modulus is related to the lower density, caused by incomplete melting in the SLM process. The existence of pores, due to incomplete melting, is observed in the S4 specimens, which is to be presented and discussed in the following sections. Table 3 shows that S4 has the lowest relative density, i.e., the largest porosity ratio. A greater porosity ratio would lead to a lower stiffness of a structure. Comparison of the average ultimate tensile stress among Groups S1 to S4 indicates it decreases with an increase in laser spot size. The Group S4 has the largest laser spot size and re-heated zone with a smaller temperature gradient, allowing more time for grain growth. Therefore, the lowest ultimate tensile strength of S4 can be attributed to a coarser microstructure with larger grain size. Although there is a significant difference in the ultimate tensile stress among the given SLM builds, their yield stress values are comparable. This is because the deformation-induced phase transformation of unstable, retained austenite to martensite might occur at a certain stress level, which corresponds to the turning point, around 200 MPa, in the stress-strain curves. In general, the tensile properties are improved with a decrease in laser spot size. Therefore, the lower density, generated by a larger laser spot, has a lower tensile strength and elongation. The microstructures of all groups are presented and discussed in Section 3.5. Note the above results represent for the as-built SLM AISI 420 parts of no machining or polishing.

### 3.4. Fractography Analysis

**Figure 4:**
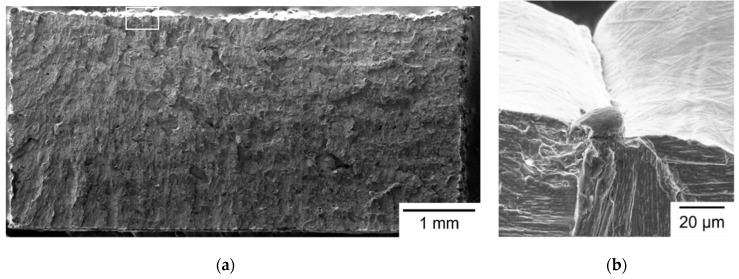
Fractography of a Group S1 specimen observed by SEM: (**a**) whole fracture surface; (**b**) fracture origin outlined in (**a**).

The typical fracture surface morphology of Group S1 specimens is shown in Figure 4. Figure 4a shows the entire fracture surface, with a flat brittle fracture pattern. Brittle fracture features, such as cleavage and river patterns, are visible at a high-magnification SEM micrograph, as shown in Figure 4b. The fracture origin can be identified by tracing the river patterns to the starting point, as shown in Figure 4b. EDS was used to identify the composition at the fracture origin and confirmed it was an inclusion with high carbon and low iron contents. The formation of an inclusion may be attributed to contamination in the manufacturing process of powder. As shown in Figure 4b, crack initiates at the top surface, which is the final layer of the SLM build. The final top layer of SLM build generally exists a larger tensile residual stress, as well as a larger surface roughness [26,30]. When the laser beam irradiates the final top layer during SLM process, a high temperature gradient leads to non-uniform expansion and contraction and induces residual stresses in the build. Hence, tensile residual stress is formed on the top layer after cooling [26,30]. The top surface is also rough, resulting from the alternating scanning path. As a result, inclusion, residual stress, and surface roughness are responsible for the fracture initiation site of such S1 specimens, shown in Figure 4.

**Figure 5:**
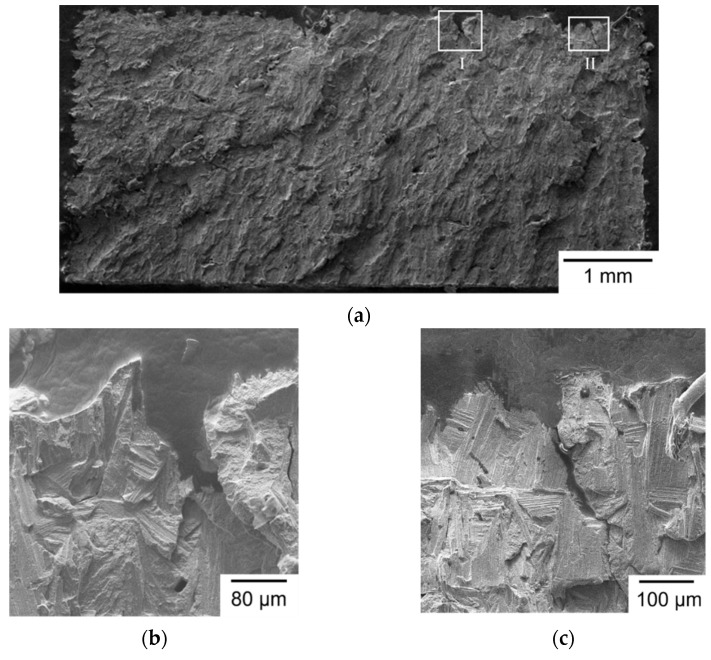
Fractography of a Group S2 specimen observed by SEM: (**a**) whole fracture surface; (**b**) fracture origin I; (**c**) fracture origin II.

A representative fracture surface of Group S2 specimens, shown in Figure 5a–c, shows the possible fracture origins in high magnification of the outlined square regions in Figure 5a. Pores and microcracks were found at the fracture origins. Pores and microcracks usually exist at the boundary between melt pool and un-melted powder. Moreover, the fracture origin is also located on the top layer of the SLM build. As a result, pores/microcracks, tensile residual stress, and surface roughness all contribute to the fracture initiation site in such S2 specimens. Again, brittle fracture features are visible in Figure 5.

**Figure 6:**
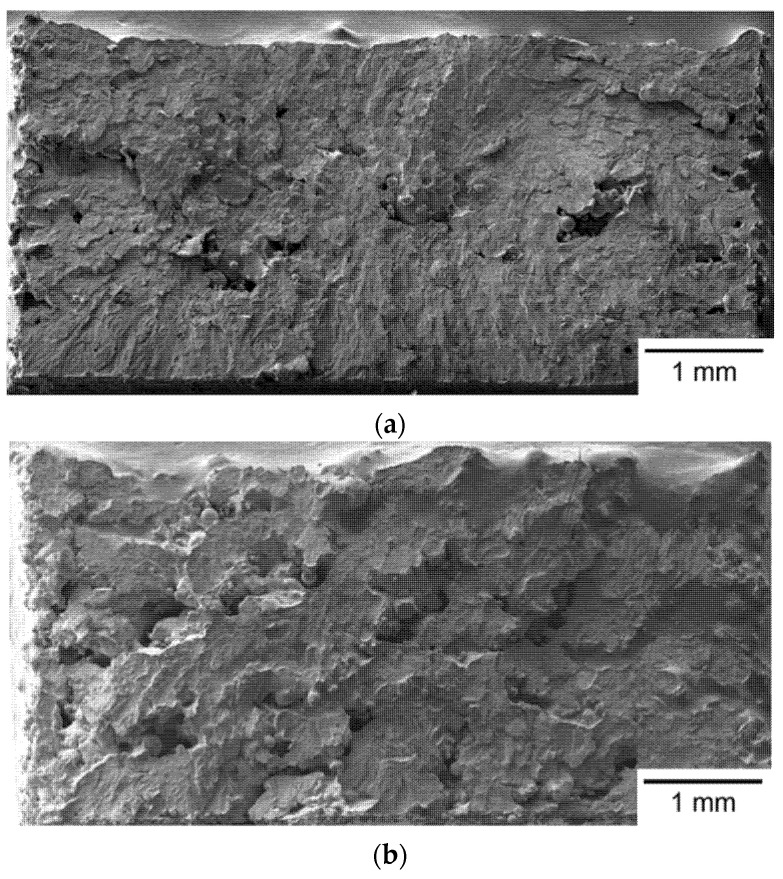
Fracture surface of (**a**) a Group S3 specimen and (**b**) a Group S4 specimen observed by SEM.

A typical fracture surface of Group S3 specimens is shown in Figure 6a. Figure 6b shows the typical fracture surface of Group S4 specimens. Both fracture surfaces also show a brittle fracture pattern. Compared to Figure 4 and Figure 5, a larger number and a greater size of pores are found on the fracture surfaces in Figure 6. The stress concentration around the pores is larger than that of other smooth regions, making such defects the favorite of crack initiation and propagation site. Therefore, the position of the fracture origin is located at the inner of the fracture surface, instead at the top edge, as shown in Figure 6a,b. The appearance of a larger number of pores on the fracture surface of S3 and S4 specimens indicates that more SLM processing defects were generated in these two groups of specimens. The existence of a larger amount of pores in S3 and S4 specimens is attributed to a larger laser spot size and a lower laser energy density applied in the SLM process. Because of the improper joining between two layers with insufficient laser energy, a lot of pores were generated in the SLM build and provided the weakest site for crack initiation and propagation. In addition, the poor Young’s modulus found in S4 specimens is also attributed to the existence of a larger number and greater size of pores.

### 3.5. Microstructural Analysis

**Figure 7:**
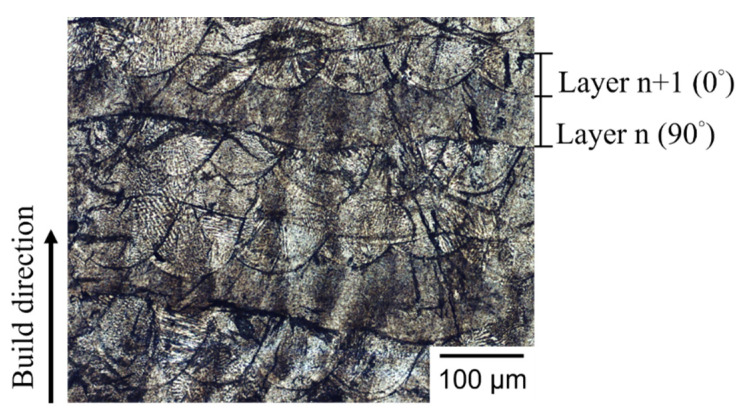
OM micrograph of typical layer morphology of SLM build.

**Figure 8:**
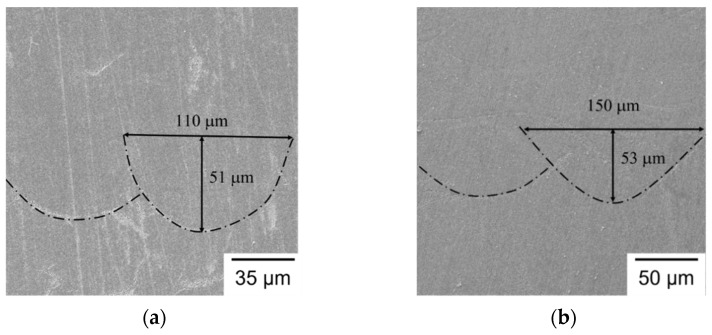

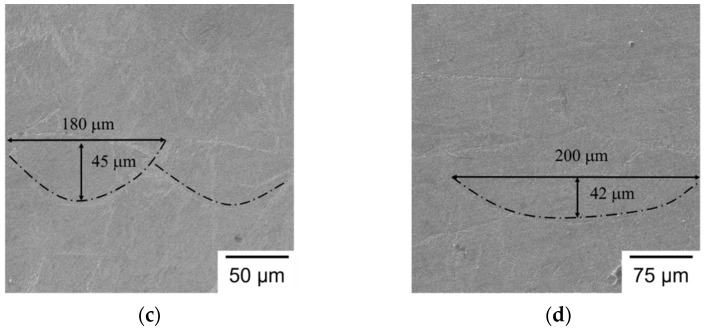
SEM micrographs of melt pool: (**a**) S1; (**b**) S2; (**c**) S3; (**d**) S4.

**Figure 9:**
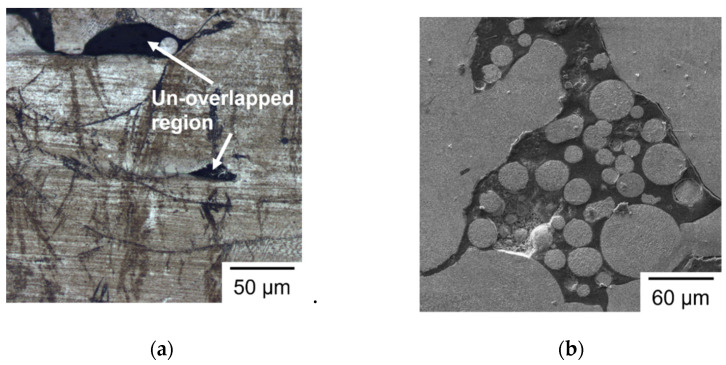
Micrographs of typical pores observed in (**a**) S3 (OM) and (**b**) S4 (SEM).

Figure 7 shows the typical layer morphology and melt pool structure of SLM builds observed by OM in a transverse cross section. As shown in Figure 7, the build direction is from bottom to top and the 90° rotation of layer scanning strategy between two successive layers is clearly seen in the layer structure. Figure 8 shows the typical transverse cross section of melt pool for different groups of specimens. As shown in Figure 8, the melt pools exhibit semi-elliptical fusion boundaries (marked by dash lines) on the observed planes. As shown in Figure 8, the depth of melt pool is 51, 53, 45, and 42 μm for Group S1 to Group S4, respectively, and the corresponding width of melt pool is 110, 150, 180, and 200 μm, respectively. In the present study, as the hatch distance remains the same in all specimens of various laser spot sizes, the overlapping area of adjacent melt tracks increases with laser spot size. Accordingly, the width of the melt pool increases with laser spot size, while the depth of melt pool slightly decreases with an increase in laser spot size. The dimensions of the melt pool in the given SLM builds clearly have a close relation with the laser spot size. It is noticed that the depths of the melt pool in Group S3 and Group S4 are less than the layer thickness setting of 50 μm. Therefore, it is expected that pores are formed inside these specimens. Figure 9 shows the typical pores observed in Group S3 and Group S4. As shown in Figure 9a, due to a smaller depth of the melt pool, the pores are formed between the melt pool boundaries and at the edge between two layers. As shown in Figure 9b, porosity is even worse and un-melted powder is also found. For S3, most of the pores exist at the un-overlapped region. For S4, most of the pores exist around the un-melted powder. As noted above, *E_V_* is 26.7 and 20 J/mm^3^ for S3 and S4, respectively. Apparently, for the given layer thickness setting of 50 μm in the SLM machine, these two lower laser energy densities are insufficient to fully melt the powder, due to a larger laser spot under a constant laser power. Accordingly, the resulting process defects are responsible for the lower density and poor mechanical properties in the S3 and S4 specimens noted above.

In our earlier study [26], martensite and retained austenite are the two main phases existing in the as-built SLM AISI 420 stainless steel. For martensitic stainless steel parts fabricated by SLM, retained austenite also exists in the as-built state, which is attributed to the high cooling rate experienced in SLM process, in a way similar to the quenching process. During the cooling process, the melt pool rapidly solidifies to form the oriented austenite (γ phase) grains, such that most of the retained austenite is nearly parallel to the build direction. When the temperature decreases to the starting point of martensite transformation (*M_s_*), the solidified melt pool is transformed into martensite (α’ phase), in a lath structure [31]. To identify the crystalline phases in the SLM builds of AISI 420 fabricated in this study, Group S1 was selected as a representative for XRD analysis, as the specimens in this group have the highest density among the given SLM builds. Note that the existing pores may affect the accuracy of XRD analysis, such that S1, having the lowest porosity, was selected. The XRD analysis results confirms that only martensite and retained austenite, indeed, exist in the as-built SLM builds of AISI 420 in this study. The volume fraction of the retained austenite was then determined using the XRD results. Details of the calculation procedure for determining the volume fraction of retained austenite were given in our earlier study [26]. In this way, the volume fraction of retained austenite in Group S1 is determined as 32%, and the remaining 68% is martensite. The content of martensite is more than twice that of austenite, resulting from a rapid cooling rate in the SLM process. During the cooling process, most of the austenite transforms to martensite at the temperature range between the martensite-start temperature and martensite-finfish temperature. However, a certain amount of austenite does not transform to martensite, so about 30% of austenite is detected in the specimens, namely retained austenite.

**Figure 10:**
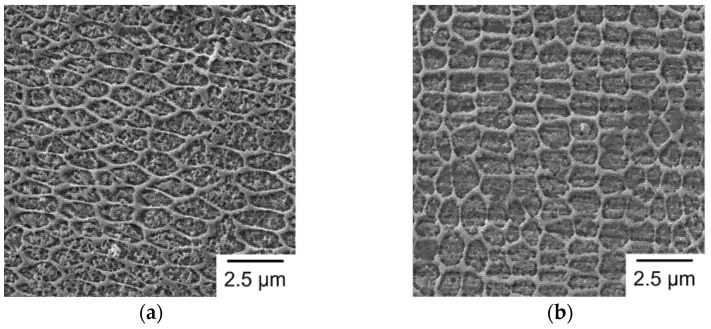

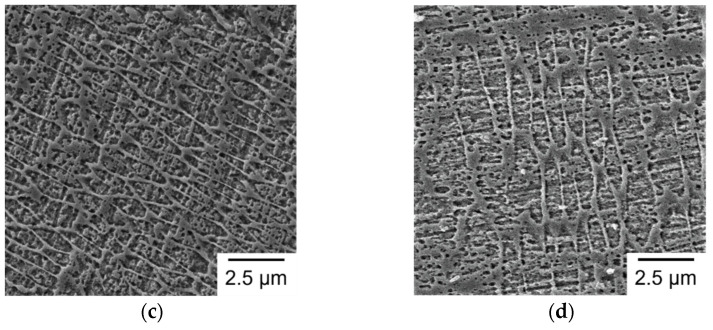
SEM micrographs of sub-grain structure in high-magnification view: (**a**) S1; (**b**) S2; (**c**) S3; (**d**) S4.

The solidification of the melt pools in SLM fabricated martensitic stainless steel was along the direction having the largest temperature gradient, usually from the boundary to the center of the melt pool. Therefore, during initial solidification of a melt pool, austenite heterogeneously nucleates at the boundary and then grows toward the center to form an austenite-phase melt pool [31]. After cooling below the *M_s_* temperature, the prior austenite phase transforms to martensite during the rapid cooling stage. As a result, elongated cellular structures are outlined by the prior austenite grain boundaries, and the martensite laths are embedded in the sub-grain structures, as shown in Figure 10. Figure 10 shows the high-magnification SEM micrographs of the elongated cellular structure within the melt pool, for each given group. As shown in Figure 10, only the retained austenite is clearly observed in the cellular structures, but the martensite laths within the cells are barely seen. The reason is that the very fine lath structure of martensite is sensitive to etching, such that it is barely seen in the etched sample. It was found that the sub-grain size is highly related to the size of laser spot (Figure 10). An increase in melt pool width and a decrease in melt pool depth for a larger laser spot size will slow down the cooling rate of the material to produce a coarser final microstructure. In addition, as the laser spot size increases, the re-heated zone also increases. Accordingly, the sub-grains of S3 and S4 are relatively elongated (Figure 10c,d), in comparison to S1 and S2 (Figure 10a,b), as they have more time to grow along the direction of solidification. For Group S4, the laser spot size is 0.4 mm, and the width of melt pool is 0.2 mm. Therefore, it has a larger re-heated zone compared to other groups. Due to the reheating of the subsequent laser beam, grains in the re-heated zone have a longer time to grow to a larger size. Consequently, a coarser microstructure is responsible for the lower mechanical strength of Group S4.

As described in Section 3.3, the given SLM built AISI 420 stainless steel exhibits a brittle fracture manner, with an elongation around 1%. This is due to the fact the major phase in the as-built SLM specimens is fine martensite, which is a typical brittle phase. In addition, the phase content in the gauge section and grip section of the specimen after tensile testing was also calculated from the XRD data of S1 specimen. Retained austenite content in the gauge section is lower than that of the grip section, by an extent of 3.57%, since more retained austenite in the highly stressed gauge section transforms to martensite during tensile testing. The reason for this phenomenon is that the gauge section is subject to a larger tensile stress, compared to the grip section, leading to a greater extent of phase transformation. This provides evidence to support the deformation-induced transformation, by which unstable retained austenite may transform to martensite during tensile testing, particularly in the gauge section.

### 3.6. Effect of Laser Spot Size

For the given SLM process conditions, the melt pools all exhibit a semi-elliptical shape, as shown in Figure 8. This corresponds to a conduction melting mode, in contrast to the keyhole melting mode of a relatively large melt pool depth [20]. It indicates the *E_V_* values (20-80 J/mm^3^), for the laser spot diameters (0.1–0.4 mm) given in the SLM process, do not exceed the critical value, *E_C_*, of over-melting. Therefore, the splashes on the surface of the final layer (observed in Figure 2) were caused by balling, and not by the spattering of over-heating with a high evaporation rate. As described in previous sections, laser spot size has proven to be an important process parameter and has significant effects on the characteristics of the SLM builds of AISI 420 stainless steel. For a given laser power, the volumetric energy density is decreased, with an increase in laser spot size. A large laser spot, with low volumetric energy density, could result in balling, un-overlapped defects, large re-heated zone, and large sub-grain size. Balling, formed by surface tension, could deteriorate the surface roughness of the given SLM builds. Un-overlapped and un-melted defects/pores degrade the density, hardness, and Young’s modulus. A large re-heated zone reduces the temperature gradient and leads to a large sub-grain size, which reduces the ultimate tensile strength. The effects of porosity, surface roughness, and defects on the mechanical properties have also been investigated for other AM-built alloys, e.g., 316L stainless steel, by SLM [32], and Ti-6Al-4V, by electron beam melting (EBM) [33]. The presence of process-induced defects, such as voids, cracks, and un-melted particles, would affect the mechanical properties of the AM parts and result in the reduction of the elongation to failure and the tensile strength [33]. In addition, fatigue failure initiates at the surface defects for AM builds subjected to low load levels, while the internal defects are responsible for the fatigue failure initiation under higher load levels [32]. Overall, it is demonstrated, in the present study, that a large laser spot with a low volumetric energy density has detrimental effects on the surface roughness, density, hardness, mechanical strength, and microstructure of the SLM builds of AISI 420 stainless steel, due to the presence of surface and internal defects. To ensure complete melting of the powder, and avoid instability of melt pool, *E_V_* of 80 J/mm^3^ seems to be a suitable laser energy density for the given SLM processing and material system.

## 4. Conclusions

(1) Laser spot size greatly influences the surface roughness of the given SLM builds of AISI 420 stainless steel. SLM builds of the largest laser spot, diameter of 0.4 mm, exhibit the roughest surface, due to a greater extent of balling. The average surface roughness, measured along the laser scanning direction, is smoother than that in the transverse direction.
(2) Densification and hardness have a good correlation. Both are affected by the porosity which is increased with an increase in laser spot size. A larger amount of pores is formed with a larger laser spot diameter, due to a lower volumetric energy density and improper joining between two layers. A SLM build of a relative density of 97% is fabricated using a laser spot diameter of 0.1 mm, with *E_V_* of 80 J/mm^3^, while a laser spot diameter of 0.4 mm, with *E_V_* of 20 J/mm^3^, produces a lower one of 91%.
(3) The stress-strain curves of AISI 420 specimens fabricated by the SLM process behave in a bi-linear manner, which is attributable to the phase transformation of unstable retained austenite to martensite during tensile testing. The SLM builds fabricated by the largest laser spot size of 0.4 mm have a relatively low Young’s modulus because of a higher fraction of porosity. The ultimate tensile stress decreases with an increase in laser spot size. Group S4 specimens, with the largest laser spot, exhibit the lowest ultimate tensile stress, due to a larger re-heated zone and coarser microstructure. Although there is a significant difference in the ultimate tensile stress among the given SLM builds with various laser spot sizes, their yield stress values are comparable.
(4) For the given SLM processing system, the melt pools all exhibit a semi-elliptical shape, corresponding to a conduction melting mode. Among the given laser spot diameters of 0.1–0.4 mm, the SLM builds of AISI 420 fabricated by a laser spot diameter of 0.1 mm, with volumetric energy density of 80 J/mm^3^, exhibit improved surface roughness, density, hardness, ultimate tensile stress, and microstructure. Accordingly, *E_V_* of 80 J/mm^3^ appears to be a suitable laser energy density for the given SLM processing and material system, so as to ensure the complete melting of the powder and to avoid the instability of the melt pool.

## Figures and Tables

**Table 1:** SLM process parameters for fabricating tensile test specimens.

Parameter	Value
Laser power (W)	400
Laser scanning speed (mm/s)	1000
Laser spot diameter (mm)	0.1 (S1), 0.2 (S2), 0.3 (S3), 0.4 (S4)
Layer thickness (μm)	50
Volumetric energy density, EV (J/mm^3^)	80 (S1), 40 (S2), 26.7 (S3), 20 (S4)
Hatch distance (mm)	0.08
Preheated temperature of baseplate (°C)	95
Scanning strategy	Island pattern with alternating path

**Table 2:** Surface roughness in *x-* and *y*-directions of SLM AISI 420, built with various laser spot sizes.

Group		*R_x_* (μm)		*R_y_* (μm)	
		Average	Standard Deviation	Average	Standard Deviation
S1	$R_{a}$	11.520	3.493	17.786	12.543
	$R_{max}$	154.584	27.646	173.839	134.267
S2	$R_{a}$	13.912	4.189	16.305	17.279
	$R_{max}$	183.800	25.495	183.800	156.425
S3	$R_{a}$	12.316	2.911	15.925	14.805
	$R_{max}$	192.829	26.829	260.721	109.078
S4	$R_{a}$	23.027	5.244	15.252	14.040
	$R_{max}$	313.609	63.008	291.453	87.331

**Table 3:** Density and hardness of SLM AISI 420 built with various laser spot sizes.

Specimen ID	Relative Density, *ρ*_r_	Hardness (HRC)
S1-1	0.96	61.2 ± 7.3
S1-2	0.97	66.8 ± 4.5
S1-3	0.97	56.9 ± 5.9
Group S1 (average)	0.97	61.7
S2-1	0.97	58.0 ± 6.9
S2-2	0.95	59.7 ± 5.4
S2-3	0.96	54.2 ± 4.0
Group S2 (average)	0.96	57.3
S3-1	0.95	54.4 ± 7.5
S3-2	0.95	55.5 ± 6.5
S3-3	0.95	58.9 ± 7.6
Group S3 (average)	0.95	56.3
S4-1	0.91	52.6 ± 6.3
S4-2	0.92	62.5 ± 4.3
S4-3	0.90	54.1 ± 3.1
Group S4 (average)	0.91	56.4

**Table 4:** Tensile properties of SLM AISI 420 built with various laser spot sizes.

Specimen ID	Young’s Modulus	Yield Stress	Ultimate Tensile Stress	Elongation
	(GPa)	(MPa)	(MPa)	(%)
S1-1	177.5	199.2	722.0	0.99
S1-2	188.6	193.1	732.3	1.02
S1-3	162.6	202.9	688.6	1.00
Group S1 (average)	176.2	198.4	714.3	1.00
S2-1	181.4	182.1	659.5	0.95
S2-2	180.0	221.5	688.6	1.01
S2-3	173.5	202.4	662.0	1.01
Group S2 (average)	178.3	202.0	670.0	0.99
S3-1	170.5	170.3	635.1	0.96
S3-2	177.1	204.8	625.8	0.95
S3-3	177.0	222.5	590.8	0.80
Group S3 (average)	174.9	199.2	617.2	0.85
S4-1	165.4	184.3	611.5	0.91
S4-2	138.0	187.9	548.1	0.90
S4-3	162.8	221.1	589.6	0.85
Group S4 (average)	155.4	197.8	583.1	0.89

## Data Availability

Data sharing not applicable.
